# Root and alveolar bone changes in first premolars adjacent to the traction of buccal versus palatal maxillary impacted canines

**DOI:** 10.1371/journal.pone.0226267

**Published:** 2019-12-10

**Authors:** Yalil Augusto Rodríguez-Cárdenas, Gustavo Armando Ruíz-Mora, Aron Aliaga-Del Castillo, Heraldo Luis Dias-Da Silveira, Luis Ernesto Arriola-Guillén

**Affiliations:** 1 Division of Oral and Maxillofacial Radiology, School of Dentistry, Universidad Científica del Sur, Lima, Perú; 2 Department of Orthodontics, Bauru Dental School, University of São Paulo, Brazil; 3 Division of Oral Radiology, Department of Surgery and Orthopedics, Federal University of Rio Grande do Sul, Porto Alegre, Brazil; 4 Division of Orthodontics and Division of Oral and Maxillofacial Radiology, School of Dentistry, Universidad Científica del Sur, Lima, Perú; Universidade Federal Fluminense, BRAZIL

## Abstract

**Objective:**

To compare the root and alveolar bone changes in first premolars adjacent to the orthodontic traction of buccal versus palatal maxillary impacted canines (MIC).

**Materials and methods:**

Before and after traction, cone beam tomographic computed (CBCTs) of 25 subjects with unilateral/bilateral MIC were included in this follow-up and retrospective study. Thirty-six first premolars were divided into 2 groups, buccal (n = 15) or palatal (n = 21) MIC, and the tomographic images were evaluated before and after orthodontic traction. Root changes in length and area were measured in sagittal, coronal and axial sections. Dimensions of alveolar bone were evaluated in coronal sections. Intergroup and intragroup comparisons were performed using t or Mann-Whitney U tests. Multiple linear regressions analyses were used to evaluate the influence of all predictor variables on root and alveolar bone changes (*P*<0.05).

**Results:**

Root and alveolar bone changes produced by orthodontic traction were not significant between groups. Root changes were smaller than 1 mm (length) and 2.51 mm^2^ (area). Alveolar bone changes between buccal and palatal MIC groups ranged from 0.13 mm to 1.69 mm Furthermore, the multivariate analysis showed no significant influence of the impaction condition (buccal or palatal) on root change. Nevertheless, some different predictor variables of the MIC influence these changes. In the alveolar bone, the maximum upper alveolar width (MUAW) is the most affected by the traction of the MIC.

**Conclusions:**

Orthodontic traction of buccal vs palatal MIC produces similar resorptive and appositional root and alveolar bone changes in the adjacent first premolars, without clinical relevance.

## Introduction

One concern after the traction of a maxillary impacted canine (MIC) is the effect produced on neighboring structures. The most frequently identified sequel is root resorption (RR), which is defined as an irreversible change that is asymptomatic but produces undesirable consequences, and it has been primarily studied in the maxillary incisors [1, 2]. However, few studies [3, 4] have examined the effect of MIC traction in the first premolars. Woloshyn *et al*.[3] used conventional radiographs and found a shortening in root length (approximately 1.27 mm) compared to the unaffected contralateral side. Silva *et al*.,[5] using only a post treatment cone beam computed tomography (CBCT), did not find significant differences in root length and alveolar bone of the first premolars after traction of unilateral palatal MIC when they compared the affected side vs unaffected.

However, the use of 3D images in this topic are limited. The reports on pretreatment cone beam computed tomographies (CBCTs) primarily focused on the prevalence of RR in neighboring teeth, and it oscillated from 2.8% to 4.48% in first premolars [4, 5]. The side effects of MIC traction were primarily studied in periodontal soft tissues,[6, 7] but the changes in the alveolar bone of premolars adjacent to the maxillary impacted canine (PAMIC) and root length and area before and after traction of MIC they have not been studied yet.

There are reasons to think that the initial location of MIC and its subsequent treatment, could affect the root of the PAMIC and its alveolar bone in a different way if it by buccal or palatal. The canine in buccal cases may be impacted against the distal surface of the lateral incisor or beyond it, and frequently its root is located at the apex of the PAMIC and reaches the upper alveolar bone zone of this teeth. Otherwise, the crown in palatal cases may contact the posterior radicular incisor surface and its root is generally close to the PAMIC root, and both are in contact in many cases. Of the teeth adjacent to the MIC, the first premolar has the largest root, and it is a pillar or immediate anchorage element that directs the traction and determines the final position of the canine in many cases.

These aspects suggest that the initial position of MIC and its traction could influence the degree of RR and the surrounding alveolar bone of the PAMIC. Premolars are responsible for 30% to 40% of the masticatory efficiency [8]. Therefore, the finding of significant effects on the PAMIC and its alveolar bone after MIC traction would be relevantly clinically important for its long-term prognosis. However, no studies evaluated this issue. Therefore, the present study compared root and alveolar bone changes of PAMIC after the orthodontic traction of buccal versus palatal maxillary impacted canines.

## Material and methods

The Ethics Committee of the Universidad Científica del Sur, Lima–Peru, approved this retrospective and follow-up study (protocol number 00007). Written informed consent was obtained for all participants and parents’ of minors and all data were fully anonymized before the researchers accessed them. This research evaluated 50 CBCTs (25 before and 25 after traction of MIC) from 25 patients treated at a private clinic (GARM). The sample (unit of analysis) consisted of 36 first premolars adjacent to permanent maxillary impacted canines that underwent traction until the occlusal plane. All MIC were classified into two groups according to location of impaction, i.e., buccal MIC (15) and palatal MIC (21) [9]. Table 1. This condition was defined on CBCT axial cuts that evaluated the following parameters: position of the MIC crown in relation to a midline drawn between the two cortical layers, and its location in relation to the neighboring lateral incisor or temporary canine.

**Table 1:** Initial characteristics of the patients according to sex and impaction canine location and evaluation of the sector of impaction in buccal or palatal MIC.

**Variable**	**Categories**	**Condition**		**Total**	**p Chi square**
		**Buccal**	**Palatal**		
Sex	Male	7	7	14	0.332
	Female	4	7	11	
	**Total (subjects)**	**11**	**14**	**25**	
Impacted canine location		**Unilateral**	**Bilateral**	**Total**	
		16	9	25	
		**Buccal**	**Palatal**	**Total**	**p Chi square**
Impaction sector of maxillary canine	Sector 1	0	2	2	0.529
	Sector 2	3	2	5	
	Sector 3	6	7	13	
	Sector 4	3	8	11	
	Sector 5	3	2	5	
	**Total (teeth)**	**15**	**21**	**36**	

A minimum sample size of 14 teeth per group was necessary to have 80% of power, to detect a difference between groups of 1.60 mm in the maximum upper alveolar width (MUAW), using a standard deviation of 2.46 mm (obtained from a previous pilot study) and with a level of significance of 0.05.

CBCTs were obtained at pretreatment (T_0_) and after orthodontic traction of MIC when the canine reached the occlusal plane (T_1_). The following inclusion criteria were used: patients older than 12 years of both sexes with buccal or palatal MIC; unilateral or bilateral impaction; PAMIC erupted, uniradicular or with roots fused into one, with complete apical closure at the beginning of the orthodontic traction; and no loss of permanent teeth. Patients with craniofacial anomalies or syndromes, periapical lesions circumscribed to the MIC at pretreatment, a history of previous orthodontic treatment, history of trauma or supernumerary teeth in the impaction zone were excluded.

The complete clinical records of each patient were registered, including demographic information, study models, intra- and extraoral photographs, panoramic and lateral radiographs and CBCTs.

**Fig 1:**
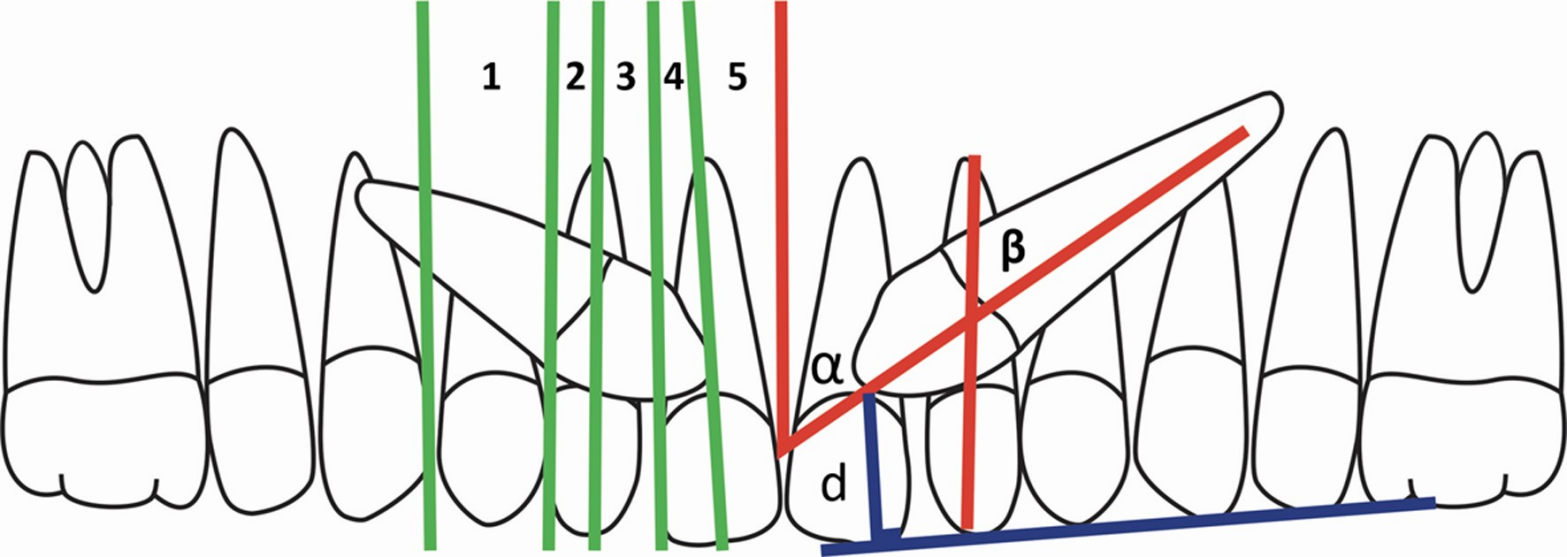
Left side: MIC position according to Ericson and Kurol.^2^ Right side: Evaluation of α and β angles and ¨d¨ distance of MIC.

Skeletal sagittal relationships (ANB[10] and APDI[11]) were evaluated on lateral radiographs. All characteristics of the MIC (sector, height, α and β angles) and the duration of orthodontic traction were also registered.[9] The diagnosis of impaction sector was applied on panoramic images synthesized from CBCTs according to Ericson and Kurol´s classification [12]. (Fig 1).

All CBCT scans were obtained using a PaX-Uni 3D (Vatech Co., Ltd., Hwaseong, South Korea) with the following parameters: 4.7 mA, 89 KVp and exposure time 15 seconds. Each field of view mode was 8 cm x 8 cm, with a voxel size of 0.2 mm. DICOM images were analyzed with Dolphin-3D software (version 11.8 Dolphin Imaging, Chatsworth, CA, USA) using multiplanar and 3D reconstructions.

### Initial root measurements

**Fig 2:**
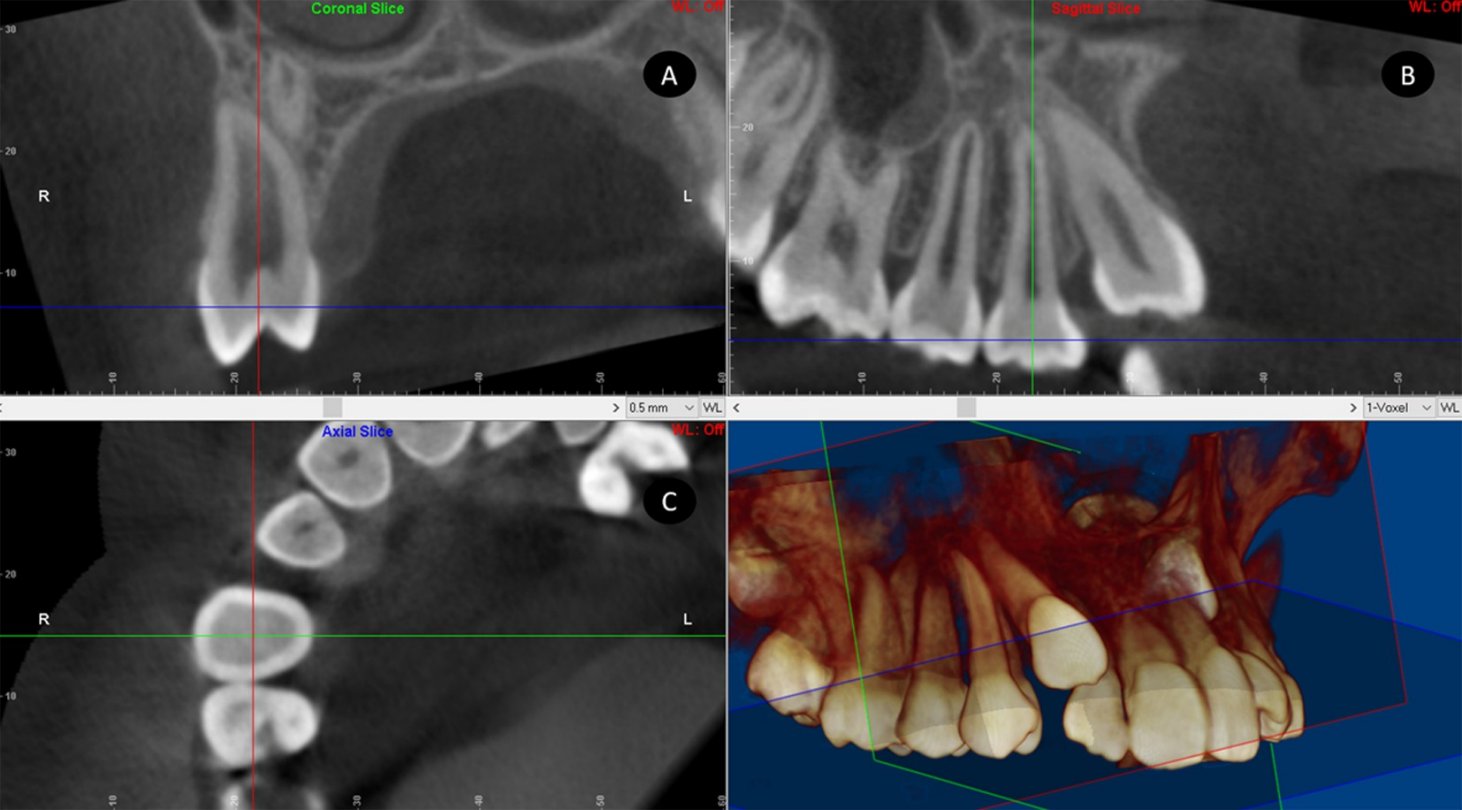
Location of PAMIC. A, coronal; B, sagittal; and C, axial sections.

**Fig 3:**
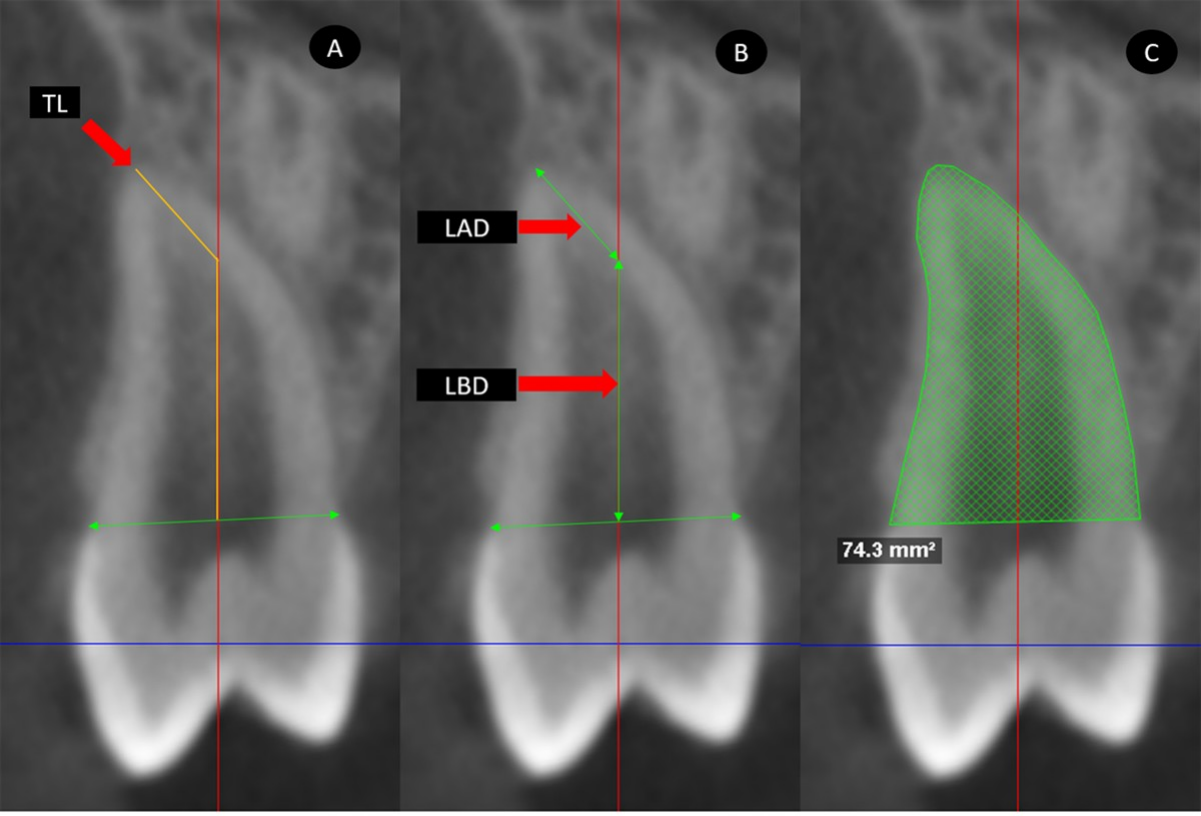
Coronal section measurements. A, total length (TL). B, length before dilaceration (LBD), length after dilaceration (LAD). C, evaluation of root area.

**Fig 4:**
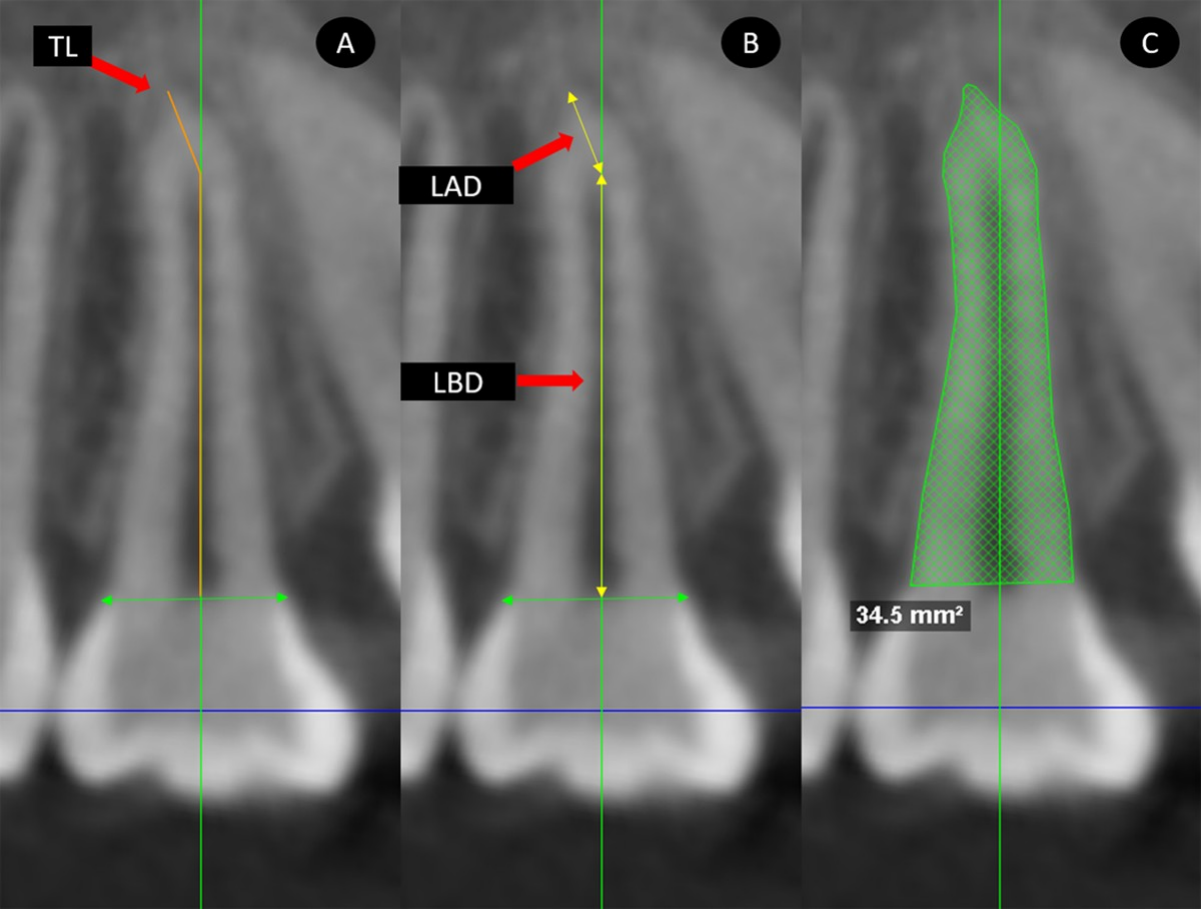
Sagittal section measurements. A, total length (TL). B, length before dilaceration (LBD), length after dilaceration (LAD). C, evaluation of root area.

DICOM images were analyzed using the same software. Coronal, sagittal and axial sections of each PAMIC were obtained. The corresponding section was aligned with the longitudinal tooth axis in the coronal and sagittal planes via locating the largest mesiodistal diameter of the premolar crown perpendicular to the sagittal plane in the coronal section and perpendicular to the coronal plane in the sagittal section (Fig 2). Root lengths were measured in mm from the center of a line that connected the buccal-palatal or mesial-distal enamel-cement junction (in the coronal or sagittal sections, respectively) to the vertex of premolar radicular apex (TL: total length). In the event of presence of any root dilaceration, the TL was measured as the sum of the root length before dilaceration LBD plus root length after dilaceration LAD (Figs 3 and 4).

**Fig 5:**
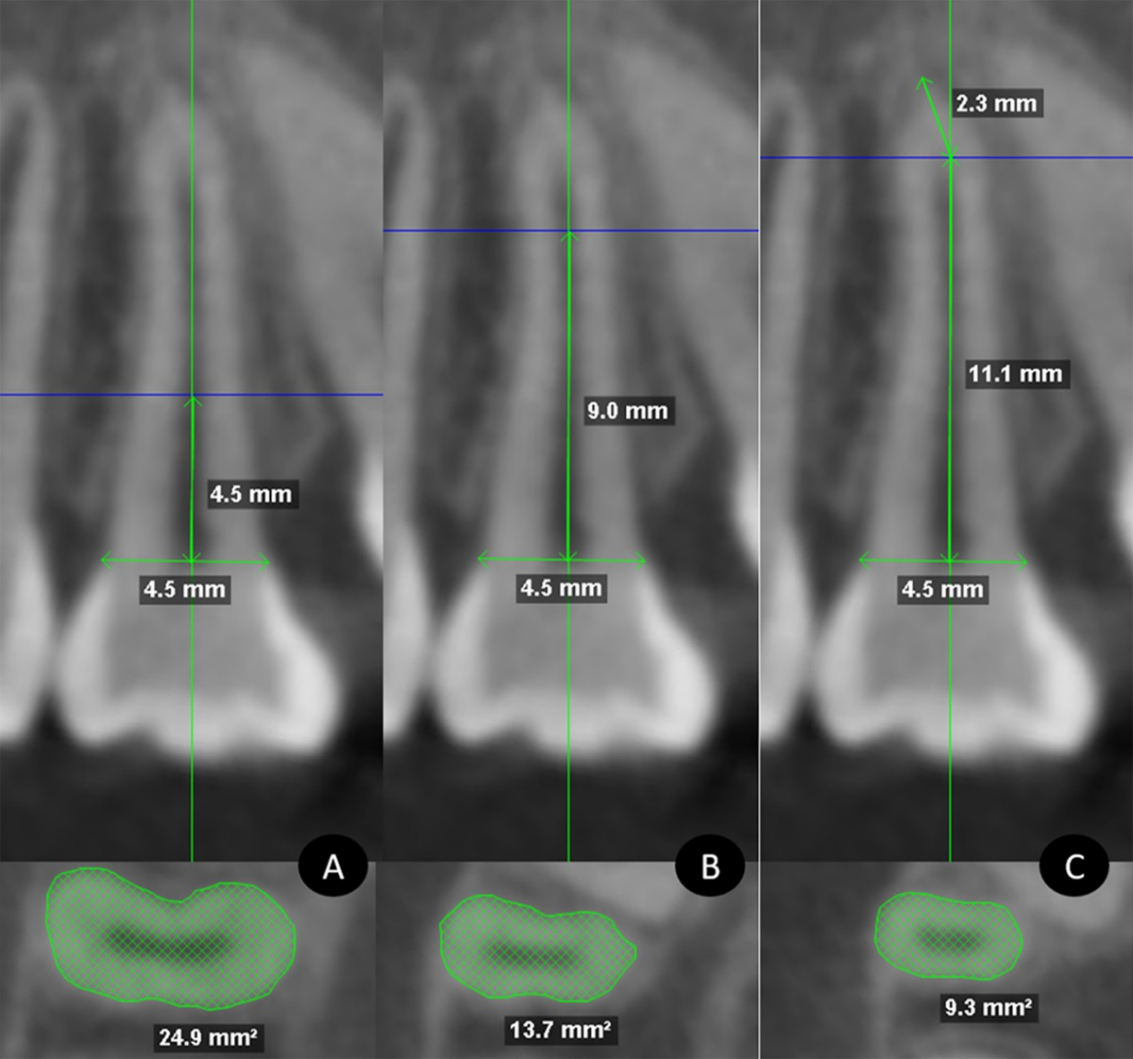
Axial section measurements. A, cervical third. B, middle third. C, region of origin of the dilaceration.

PAMIC root areas in mm^2^ were evaluated beginning from the buccal enamel-cement junction along the contour of the entire root until the palatal enamel-cement junction in the coronal section, and from the distal enamel-cement junction along the root contour until the mesial enamel-cement junction in the sagittal section. Root areas in axial views were measured at three sectors. Sectors were defined by dividing the total root length of the sagittal section into thirds. The areas at the upper limit of the cervical and middle thirds and the area of the root zone of dilaceration origin were measured in the axial sections. (Fig 5).

### Initial measurement of the alveolar bone

**Fig 6:**
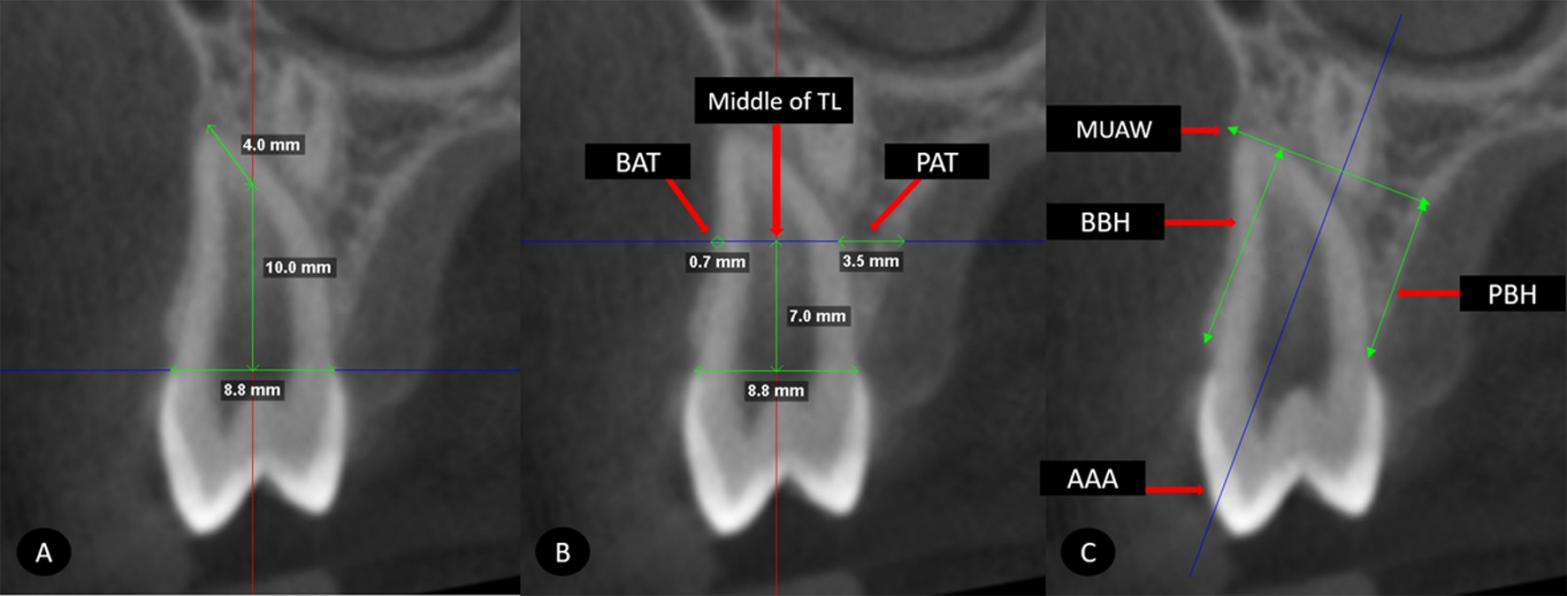
Measurements of alveolar bone. A, Location of PAMIC. B, (Middle of TL), buccal alveolar thickness (BAT) and palatal alveolar thickness (PAT). C, location of the alveolar axial axis (AAA), maximum upper alveolar width (MUAW), buccal bone height (BBH) and palatal bone height (BPH).

The premolar was aligned with the axial axis of each tomographic section. Buccal alveolar thickness (BAT) and palatal alveolar thickness (PAT) were measured from the outermost root surface of each side to the outermost surface of the palatal and buccal cortical bones, respectively. This same section was aligned with the alveolar axial axis (AAA), and a perpendicular line representing the maximum upper alveolar width (MUAW) was drawn. The perpendicular distances from MUAW to the edge of the premolar bone crest were measured on the buccal (buccal bone height, BBH) and palatal sides (palatal bone height, PBH) (Fig 6). Table 2.

**Table 2:** Definitions of the measurements used in this study.

Variables	Definition
**Skeletal sagittal relationship parameters**	
ANB	The angle between points A, N and B in degrees.
APDI	The anterior-posterior dysplasia indicator was obtained from the algebraic sum of the angles N-Pg-FH (Facial Plane) plus/minus the angle AB- Facial Plane (is positive when the point B is ahead of point A and is negative when the point A is ahead of point B) and plus/minus the angle FH-PP (palatal plane) (is negative when PP is tilted upward and positive when tilted down).
**Sagittal parameters of position and maxillary size**	
SNA	The angle between points Sella (S), Nasion (N) and Sub nasal (A) in degrees.
Maxillary length	Distance between the anterior nasal spine (ANS) and posterior nasal spine (PNS).
**Root parameters of PAMIC**	
LT	Total length: distance from the center of a line that connected the vestibular-palatal or mesial-distal enamel-cement junction until the vertex of premolar radicular apex on the axial axis of the tooth in the coronal and sagittal section. With presence of dilaceration, was measured as the sum of the root length before dilaceration (LBD) and root length after dilaceration (LAD).
Areas	Five areas in mm^2^ were measured: the coronal, from the buccal enamel-cement junction, along the contour of the entire root until the palatal enamel-cement junction; sagittal, from the distal enamel-cement junction along the root contour until the mesial enamel-cement junction; and three axial root areas: in the upper limit of the cervical and middle third, and in the root zone of dilaceration.
**Alveolar bone parameters of PAMIC**	
BAT	The buccal alveolar thickness was measured in coronal section, from the outermost root surface to the outermost surface of the buccal cortical bone, on a horizontal lines at the middle of the total length (TL), parallel to another line built from the buccal enamel-cement junction until the palatal enamel-cement junction and perpendicular to the axial axis line.
PAT	The palatal alveolar thickness was measured in coronal section, from the outermost root surface to the outermost surface of the palatal cortical bone, on the same horizontal line in which BAT was measured.
MUAW	The maximum upper alveolar width was drawn and measured in the widest alveolar area, from the outermost point of the buccal and palatal cortex, perpendicular to the alveolar axial axis (AAA).
BBH	The buccal bone height was the perpendicular distance from MUAW to the edge of the premolar bone crest on the buccal side.
PBH	The palatal bone height was the perpendicular distance from MUAW to the edge of the premolar bone crest on the palatal side.

### Canine traction technique

**Fig 7:**
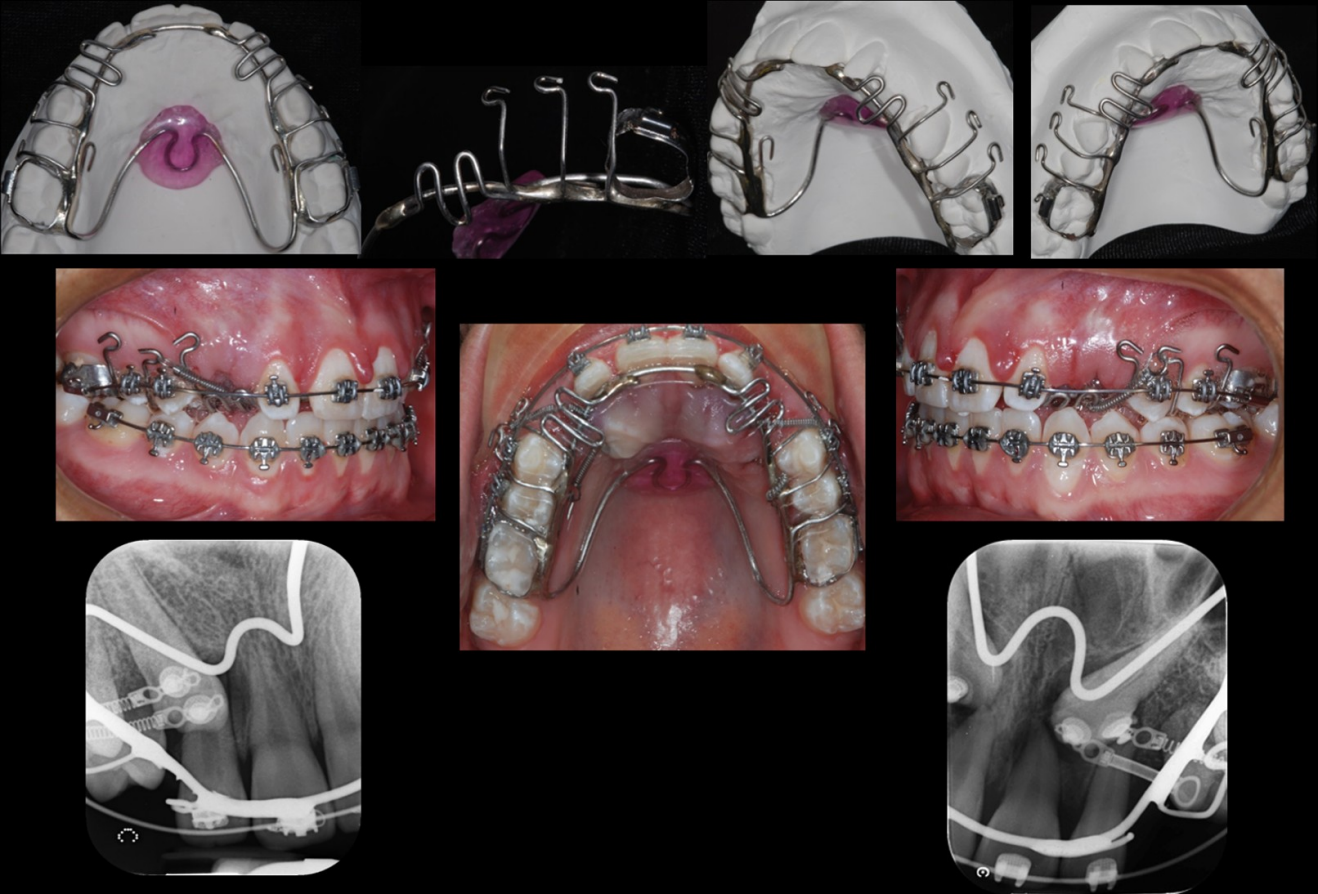
Rigid Anchorage appliance used for MIC traction.

Traction was performed following a strict orthodontic protocol in all cases using an individualized rigid anchorage device that included an acrylic palatal button soldered onto first permanent molar bands and multiple palatal-occlusal-vestibular soldered hooks of 0.028" stainless steel wire. (Fig 7). The surgical technique selected was closed,[13] but exceptionally an open technique was necessary [14]. The orthodontic treatment included bracket slots of 0.022" x 0.028" (Synergy RMO, Inc., Rocky Mountain Orthodontics Denver, Colorado, USA). The buccal hooks of the anchor were used to fasten the buckles of NiTi closed coil springs 0.010 "x 0.036", 13 mm long and 150 g force (Dentos Inc. Daegu, Korea) to perform intraosseous transalveolar traction until the MIC reached the occlusal plane. CBCTs (T1) were taken at this moment using the same technical characteristics of the initial CBCT to control the treatment and supervise the RR of maxillary incisors [15, 16]. All of the necessary procedures to complete the orthodontic treatment were performed.

### Final measurement of roots and bone changes

The root lengths and area and alveolar bone variables on this second CBCT (T_1_) were measured in the same sections. To measure changes in each PAMIC and the surrounding alveolar bone, the final value (T_1_) was subtracted from the initial value (T_0_). Positive values of the difference indicated resorptive changes, and negative values indicated appositional changes.

### Reliability

Three orthodontists performed the diagnosis of impaction. Interobserver diagnostic and positional agreement was assessed using the Kappa coefficient. Values greater than 0.9 were obtained. The primary evaluator for quantitative variables repeated their measurements after a 30-day interval. The intraclass correlation coefficient (ICC) was used to evaluate intraobserver agreement. All values were greater than 0.9. Random errors were calculated using Dahlberg’s formula, and the results were smaller than 1 mm or 1 mm^2^.

### Statistical analyses

Statistical analyses were performed using SPSS for Windows (version 19.0; IBM, Armonk, NY, USA). Descriptive statistics of root changes in mm and area in mm^2^ of each first premolar adjacent to the traction of buccal or palatal MIC were calculated. Data normality was determined using the Shapiro-Wilk’s test. Intergroup comparisons were performed using t or Mann-Whitney U tests, depending on data normality. Multiple linear regression models were used to evaluate the influence of each variable on root and alveolar bone changes, considering all of the variables as predictors (overfit method). Statistical significance was set at *P*<0.05 for all tests.

## Results

The sample initial characteristics are summarized in Table 3. No significant differences were found in intra- or intergroup comparisons of root changes of PAMIC in coronal, sagittal and axial sections. The changes in millimeters and areas between groups were smaller than 1 mm and 2.51 mm^2^ in both groups (Table 4) **S1 Database.**

**Table 3:** Initial characteristics of the sample according to impaction condition of maxillary canine.

Measurements	Impaction condition				p	Mean difference	Lower limit CI—95%	Upper limit CI—95%
	Buccal = 15		Palatal = 21					
Age*	14.27	3.47	21.05	7.55	0.009	-6.78	-11.78	-1.85
ANB	4.04	2.04	3.37	2.81	0.49	0.66	-1.29	2.63
APDI*	78.91	4.84	84.93	4.97	0.003	-6.02	-9.77	-2.27
SNA*	81.56	4.81	86.79	4.34	0.004	-5.23	-8.65	-1.80
Maxillary length ANS–PNS	48.88	3.21	50.21	4.37	0.383	-1.32	-4.38	1.73
Height of impacted canine*	12.92	3.33	8.70	1.88	<0.001	4.22	2.37	6.07
Angle α of impacted canine	47.85	19.41	43.18	14.00	0.427	4.67	-7.17	16.52
Angle β of impacted canine	49.63	25.29	43.12	13.28	0.34	6.51	-7.10	20.22

*Statistically significant at *P*<0.05

*t* test

**Table 4:** Comparison of root length and area changes (T0-T1) of PAMIC between buccal and palatal MIC groups.

CBCT section	Measurements 6		Impaction condition of maxillary canine				Mean difference	Confidence interval to 95%		P
			Buccal (n = 15)		Palatal (n = 21)					
			Mean	SD	Mean	SD		Lower limit	Upper limit	
Coronal	Root change in mm (TL)		0.21	1.00	0.81	1.56	-0.60	-1.74	0.53	0.288
	Root area change in mm^2^		1.84	8.10	1.35	9.97	0.48	-7.11	8.07	0.897
	Root change in mm (LAD)		0.48	0.70	0.57	3.59	-0.08	-3.03	2.86	0.951
	Root area change in mm^2^		1.41	2.48	-0.43	4.47	1.84	-1.95	5.64	0.115
Sagittal	Root change in mm (LBD)		0.32	1.38	0.68	3.53	-0.35	-3.34	2.64	0.806
	Root change in mm (TL)		0.71	0.98	0.80	1.12	-0.09	-0.97	0.79	0.836
	Root area change in cervical third in mm^2^		-0.83	2.51	-2.50	3.81	1.75	-1.02	4.52	0.205
Axial	Root area change in middle third in mm^2^		-0.80	4.60	-1.16	3.76	0.36	-2.99	4.82	0.825
	Root area change in curve of dilaceration in mm^2^		0.80	4.13	-1.87	9.06	2.67	-5.12	10.46	0.478

PAMIC, *premolars adjacent to the maxillary impacted canine;* MIC, *maxillary impacted canine;* LAD, *length after dilaceration*; LBD, *length before dilaceration*; TL, *total length*.

*t* test

Alveolar bone changes of PAMIC in thickness, width and height were not significantly different in intra- or intergroup comparisons. Changes between buccal and palatal MIC groups ranged from 0.13 mm to 1.69 mm (Table 5).

**Table 5:** Comparison of alveolar bone changes (T0-T1) of PAMIC between buccal and palatal MIC groups.

CBCT section	Measurements	Impaction condition of maxillary canine				Mean difference	Confidence interval to 95%		P
		Buccal (n = 15)		Palatal (n = 21)					
		Mean	SD	Mean	SD		Lower limit	Upper limit	
	Buccal alveolar thickness (BAT) †	0.28	0.98	0.13	0.62	0.15	-0.48	0.79	0.621
Coronal	Palatal alveolar thickness (PAT) †	1.06	1.20	1.69	1.27	-0.62	-1.72	0.47	0.250
	Maximum upper alveolar width (MUAW) †	1.50	1.98	1.15	1.57	0.34	-1.10	1.78	0.628
	Buccal bone height (BBH) ‡	0.35	1.75	0.99	2.04	-0.63	-2.28	1.00	0.743
	Palatal bone height (PBH) †	-0.29	1.7	1.05	2.78	-1.33	-3.43	0.75	0.200

† *t* test

‡ Mann-Whitney U test

The linear regression models, considering root changes as outcome variables, did not significantly influence the impaction condition (buccal or palatal) on root changes. However, the ANB angle (*P* = 0.034) and duration of traction (*P* = 0.010) significantly influenced the total length (TL) change in the sagittal section. APDI (*P* = 0.008) significantly influenced the changes in root area in the upper limit of the cervical third in the axial section, and age (*P* = 0.047) significantly influenced the root area change in the upper limit of the middle third of the axial section (Table 6).

**Table 6:** Linear regression model to evaluate the influence of the predictor variables in the root changes of total length at sagittal section and root area changes at sagittal, and cervical and middle third at axial sections of PAMIC.

**Predictor Variables**	**Total length (TL) in section sagittal (in mm)**	
	**B**	**P**
(Constant)	2.418	0.657
ANB	-0.461	**0.034** *
APDI	-0.033	0.612
Duration traction	0.204	0.010*
Impaction condition	-0.437	0.380
Impaction sector	0.391	0.063
**R**^**2**^	0.479	
**Predictor Variables**	**Root area changes in upper limit of the cervical third of axial section (in mm^2^)**	
	**Β**	**P**
(Constant)	-61,591	0.016
Age	-0.329	0.063
APDI	0.628	0.008*
Maxillary length	0.257	0.279
Impaction condition	-2.796	0.113
Alfa angle	0.043	0.395
**R**^**2**^	0.368	
**Predictor Variables**	**Root area changes in upper limit of the middle third of axial section (in mm^2^)**	
	**Β**	**P**
(Constant)	-59.805	0.164
Age	-0.566	0.047*
APDI	0.610	0.087
Maxillary length	0.420	0.297
Height impacted canine	-0.141	0.722
Alfa angle	-0.030	0.795
Complexity traction (α >40°)	-0.193	0.942
**R**^**2**^	0.367	

* Statistically significant at *P*<0.05.

Similarly, the linear regression models showed no significant influence of the impaction condition (buccal or palatal) on alveolar bone changes. However, buccal alveolar thickness (BAT) was significantly influenced by the height of the MIC (*P* = 0.037). Palatal alveolar thickness (PAT) was influenced by the APDI (*P* = 0.043). The maximum upper alveolar width (MUAW) was significantly influenced by sex (*P* = 0.001), SNA (*P*<0.001), ANB (*P* = 0.011), APDI (*P* = 0.017), maxillary length (*P*<0.001), duration of traction (*P* = 0.003), and impaction sector (*P* = 0.002). Buccal bone height (BBH) was significantly influenced by the ANB (*P* = 0.007) and β angle (*P* = 0.033). Palatal bone height (PBH) was influenced by age (*P* = 0.001), maxillary length (*P* = 0.001) and impaction sector (*P* = 0.034) (Table 7).

**Table 7:** Influence of predictor variables with P values smaller than 0.030 in the alveolar bone changes of PAMIC at coronal section (in mm).

**Predictor Variables**	**Buccal alveolar thickness (BAT)**	
	**Β**	**P**
(Constant)	1.920	0.021
Impaction condition	-0.692	0.089
Height impacted canine	-0.120	**0.037** *
**R**^**2**^	0.182	
**Predictor Variables**	**Palatal alveolar thickness (PAT)**	
	**Β**	**P**
(Constant)	12.882	0.026
APDI	-0.130	**0.043** *
Impacted condition	1.020	0.222
Height impacted canine	0.036	0.774
Beta angle	-0.029	0.146
**R**^**2**^	0.248	
**Predictor Variables**	**Maximum upper alveolar width (MUAW)**	
	**β**	**P**
(Constant)	7.557	0.660
Sex	-1.776	**0.001** *
SNA	0.245	**<0.001** *
ANB	-0.555	**0.011** *
APDI	-0.155	**0.017** *
Maxillary length	-0.261	**<0.001** *
Duration of traction	0.245	**0.003** *
Impaction sector	-0.613	**0.002** *
**R**^**2**^	0.888	
**Predictor Variables**	**Buccal bone height (BBH)**	
	**β**	**P**
(Constant)	-5.457	0.548
Sex	-1.247	0.288
SNA	0.072	0.470
ANB	-1.074	0.007*
Duration of traction	0.314	0.112
Height impacted canine	-0.469	0.094
Beta angle	0.110	0.033*
**R**^**2**^	0.477	
**Predictor Variables**	**Palatal bone height (PBH)**	
	**β**	**P**
(Constant)	21.145	<0.001
Age	0.235	**0.001** *
Maxillary length	-0.445	**0.001** *
Impaction sector	-0.770	**0.034** *
**R**^**2**^	0.587	

* Statistically significant at *P*<0.05.

## Discussion

The present study compared root and alveolar bone changes of the first premolars adjacent to the orthodontic traction of buccal versus palatal MIC and determined which factors affected these changes. We use a reproducible method that described its morphology in three planes of space and used measurements of length and areas. This method was previously used in two studies to evaluate the RR of incisors after the traction of the unilateral vs bilateral MIC[16] and according to its complexity [15]. The findings of similar studies cannot be compared with ours because of the differences in the radiographic technique and the methodology used. In the study by Woloshyn *et al*.,[3] they used periapical radiographs, and in the retrospective study by Silva *et al*.,[5] the bone changes were compared with the unaffected side, on CBCT post-treatment of unilateral palatal MIC, and this is not optimal. Therefore, to compare the changes before and after the traction of the same side according to the location of the MIC is the central objective of the present article.

This report is the first study to establish these three-dimensional comparisons in the first premolars after orthodontic traction of MIC. It is important to clarify that the CBCTs used in this study were required to visualize the consequences of the traction on MIC and on the non-impacted teeth at the end of MIC traction. This evaluation is based on the statement by the American Academy of Oral and Maxillofacial Radiology[17], which recommends tomographic supervision according to the complexity of the case and the need for follow up of possible RR and the undesirable effects of orthodontic traction on neighboring structures. The CBCT indicates when 2D images are not sufficient [15, 16, 18].

The sample in the present study was limited exclusively to uniradicular premolars because of the great difficulty in the standardization of a reliable method of measurement in a sample composed of biradicular or three-radicular PAMIC. However, we achieved the minimum required sample size. Furthermore, the prevalence of uniradicular premolars is remarkable. Abella *et al*.[19] reported in their study a prevalence of uniradicular of 46% in the Spanish population, but mentions that it has been reported up to 60% in the Chinese population.

This aspect allows us to extrapolate our results with almost half of the population, even further if we take into account that we include in the sample premolars with roots fused into one. Our results do not reflect all of the possible evaluations of root morphology, but it provides an approximation of the tissue response to the traction of MIC on the first premolar and the surrounding alveolar bone.

The intergroup comparisons did not show significant differences for the root or alveolar bone changes of PAMIC after MIC traction. Our hypothesis suggests that the PAMIC suffers greater root resorption and alveolar changes depending on the condition of MIC (buccal or palatal) and as a consequence of the traction process. This idea was based on some factors, such as the different bone configurations between these two conditions, different eruption direction between buccal and palatal MIC to a probable friction between roots in the traction process, and the typical morphology of PAMIC. However, differences between groups were not observed. The results in both groups showed primarily resorption, which has not become clinically relevant because it does not exceed 1 mm of length or 2 mm^2^ of area. The presence of negative values in these analyses, primarily root area change in the cervical and middle third, indicates and appositional root changes in PAMIC. More studies using a similar methodology should be performed to confirm our results.

Multiple regression analysis showed a significant influence of some skeletal sagittal variables (ANB and APDI) and maxillary size and position (SNA) on the root change of PAMIC caused by the orthodontic traction of the MIC at the level of total length in the sagittal section and the area of ​​the limit of the cervical third in the axial section and on the alveolar bone in PAT, BBH, PBH and mainly in MUAW. Our findings show great sensitivity in this last area because most variables were considered in this study, which were responsible for 88% of this change. These findings show the changes in maxilla dimensions when a canine is impacted and reflect a great dynamic in this area, which was likely due to the contact between the roots of the MIC and the PAMIC during traction.

The present analysis also showed that a longer the duration of traction produced more RR in the sagittal LT and more bone loss in the MUAW. More RR in an older patient will have the axial area in the middle third of the PAMIC and more bone loss in PBH. More bone loss at the BBH level will be present as the beta angle increases. MUAW and PBH will be more affected by the traction if the MIC is further away from the middle line because the traction will have to traverse a longer distance; The BAT tended to decrease with traction when the MIC was farther from the occlusal plane because its small dimensions focused on the behavior of the buccal cortical. These important findings should be considered in the initial planning and prognosis of buccal or palatal MIC treatment, and the results justify future studies specifically focused on these aspects.

## Conclusions

- The traction of a buccal vs palatal MIC produces similar changes in the root and the alveolar bone of the PAMIC of resorption and apposition, but these alterations are not significant.
- The orthodontic traction of MIC is not a risk for radicular integrity and alveolar bone support of the first premolars adjacent to MIC.

## Supporting information

S1 Database Premolars. (XLSX)
